# Single nuclei/cell transcriptomics reveal DMD driven cell dynamics and mechanisms of fibroblast inflammatory tissue priming in human dystrophic muscle

**DOI:** 10.21203/rs.3.rs-4934147/v1

**Published:** 2026-04-08

**Authors:** Kevin N. Chesmore, Florian Barthélémy, Sophie Rengarajan, Deirdre D. Scripture-Adams, Annabel Sen, Daniela Nasif, Isaiah Little, Richard T. Wang, Shirley Nieves-Rodriguez, Rosario I. Corona, Nicole Steady, Artemis Yip, Emilie D. Douine, Stanley F. Nelson, M. Carrie Miceli

**Affiliations:** 1 Center for Duchenne Muscular Dystrophy at UCLA, Los Angeles, CA, USA.; 2 Department of Microbiology, Immunology, and Molecular Genetics, David Geffen School of Medicine and College of Letters and Sciences, University of California, Los Angeles, Los Angeles, CA, USA.; 3 Department of Human Genetics, David Geffen School of Medicine, University of California, Los Angeles, Los Angeles, CA, USA.; 4 Department of Neurology, David Geffen School of Medicine, University of California, Los Angeles, Los Angeles, CA, USA.; 5 Institute for Precision Health, David Geffen School of Medicine, University of California, Los Angeles, Los Angeles, CA, USA.; 6 Department of Pathology and Laboratory Medicine, David Geffen School of Medicine, University of California, Los Angeles, Los Angeles, CA, USA.

## Abstract

Single cell/nuclei technologies have revolutionized our understanding of the remodeling of complex multi-cellular tissue that accompanies injury, regeneration, and disease. Duchenne muscular dystrophy (DMD) is a fatal genetic disease of childhood characterized by progressive skeletal muscle weakness resulting from mutation of *DMD* and loss of functional dystrophin. Here we report, at single nuclei/cell resolution, on intramuscular cell and gene expression dynamics within a broad cohort of needle muscle biopsies obtained from DMD individuals with varying degrees of severity, including a subset with low levels of dystrophin. We report a strong negative correlation between expression of dystrophin and disease severity and report substantial differences in cellularity and cell type-specific gene expression in DMD severity groups versus healthy muscle. Expression signatures indicate that DMD myofibers become immunologically alert, upregulating innate and adaptive immune sensors, including TLR4, IL15, TNF family receptors and MHC and costimulators. In this cohort, dystrophic muscle was remodeled with 50% fewer myofibers with expansion and diversification of fibroblasts and myeloid cells. We identify a DMD-specific TNFα-responsive Thy-1^+^/C3^+^ fibroblast subpopulation which we propose are inflammatory tissue priming fibroblasts and three DMD-specific myeloid populations which express signatures of innate immune memory. There is an 8-fold increase in CD8^+^GZMK+/GZMB+ T cells (Tek), with characteristics of both adaptive and innate immune activity. We propose that these non-myofiber muscle resident cells interact and epigenetically instill long-term tissue memory to perpetuate and amplify a hyper-inflammatory state in DMD muscle, contributing to impaired regeneration, myofiber death and fibrosis. This compendium of single/cell nuclei serves as a valuable reference and has immediate impact for biomarker discovery, clinical trial design, identification of barriers to dystrophin replacement therapies and novel druggable cell mechanisms operating in DMD.

## ONE SENTENCE SUMMARY

Single nuclei and cell transcriptomics of human DMD and healthy muscle reveal new cell types, innate immune memory, and key cell-cell signaling.

## INTRODUCTION

Duchenne muscular dystrophy (DMD) is caused by *DMD* mutations with loss of dystrophin in satellite cells and myofibers, resulting in propensity to myofiber injury, failed regeneration, progressive muscle weakness, and premature death from respiratory and/or cardiac failure. Healthy skeletal muscle is cellularly complex and plastic, responding to local injury by recruiting and expanding immune cells, fibroblasts, and other muscle resident cells to guide satellite cell-mediated repair of damaged multinucleated myofibers ^1^. The lack of dystrophin initiates persistent myofiber damage which results in immune infiltration/activation and dysregulation of repair with fibro-fatty replacement. Our understanding of the cellular and molecular mechanisms of DMD pathogenesis remains incomplete, and the study of human diseased muscle tissue is essential. Because myofibers are multinucleated, they are not conducive to characterization by single-cell analysis. Therefore, we developed protocols to minimally invasively biopsy muscle ^2^ and purify nuclei and cells from small specimens with intact cell-cell interaction ^3^. These protocols facilitate simultaneous single nuclei sequencing of multinucleated and mononuclear muscle resident cells and expand on smaller prior datasets of single cell human muscle transcriptomics ^3, 4, 5, 6, 7, 8, 9, 10, 11, 12, 13, 14, 15, 16, 17, 18, 19, 20, 21^.

Here we report on a heterogeneous cohort of muscle biopsies from DMD and healthy individuals, creating 134,943 nuclear transcriptomes. These transcriptomes are linked to clinical data, dystrophin expression in myofibers, *DMD* mRNA from bulk whole muscle transcriptomics and coupled with complementary single-cell analysis of immune mononuclear cells, including assessment of T cell surface protein marker expression coupled to transcriptomics. Matched biopsy-derived cell cultures, plasma, and frozen biopsy resources enhance interpretations. Rather than focus on a deep assessment of a few samples or cell types, we chose to broadly, and in a relatively unbiased manner, survey all muscle resident cell populations in DMD across a range of *DMD* mutations, disease severity, and ages, recognizing that this is a snapshot in the progression of disease in each individual. Further, differential gene expression across this diverse cohort, restricted to individual cell types, more powerfully identifies DMD-specific pathway alterations within satellite cells, immune cells, endothelial cells, myofibers, and fibroblasts. Gene set enrichment analyses and inferred cell-cell interactions altered in dystrophic muscle suggest an important role for inflammatory tissue priming fibroblasts within DMD muscle and highlight potential key inter-cellular communications that orchestrate persistent tissue pathology. We identify signatures of innate immune memory within DMD muscle resident fibroblast and monocyte subpopulations and potential roles for TNF/TNFR family, chemokine receptor/ligand, TLR4/TLR4agonist IL15-IL15Rα/receptor and C3/GZMK+ interactions in conditioning the DMD skeletal muscle niche for maladaptive inflammation, myofiber destruction, and fibrosis. Identified DMD gene expression signatures across published spatial transcriptomics performed in the mdx mouse model, wherein spatially discrete dystrophic foci enables association of gene expression with areas of disease pathology, confirms findings of the snRNAseq data set with selective co-localization of inflammatory tissue priming fibroblasts, trained monocytes, and Tek as well as gene expression signatures of myofiber death, TLR4 agonist and TLR4 activation to areas of dystrophic/fibrotic lesions in mdx muscle.

This single cell/nuclei atlas of human DMD is a rich community resource for identifying biomarkers mapped to specific cell sources and reveals molecular and cellular mechanisms driving tissue remodeling in DMD relative to healthy muscle with multiple implications for clinical trial design and biomarker discovery, and for identification of novel therapeutic targets.

## RESULTS

### Nuclear transcriptomes from DMD and healthy reveal cell type shifts in skeletal muscle

We collected frozen muscle biopsies, from 27 DMD individuals at ages 3–22 years. For each individual, we calculated an overall severity score, intended to reflect how severe that patient’s disease course was over their observed lifetime relative to “average DMD” and based on Northstar Ambulatory Assessments (NSAA) performed between ages 4–16 years ^22^ (Source Data 1f). We grouped DMD individuals into 3 groups reflecting relative disease severity observed at the age of biopsy as compared to a large reference set of DMD ^22^ : “Severe” indicating the most severe 3 deciles (n=6), “Typical” indicating the middle deciles (n=12), and “Mild” indicating those that are in the 3 most mild 3 deciles (n=9). None of the DMD subjects were on an exon skipping therapy, experimental therapy, or had received gene therapy. 25 of 27 were on chronic steroids, a standard therapy for DMD, and 25 of the individuals had a nonsense or frameshift mutation. Although these 25 individuals have *DMD* mutations predicted to result in no dystrophin, some individuals may have expression of low levels of dystrophin protein often generated by exon skipping resulting in reframing ^23, 24^. Thus, all biopsies were analyzed by mRNA sequencing at a mean depth of about 1,000 reads which allowed sensitive assessment of splicing resulting and determination of reading frame of *DMD* mRNA, and this was coupled with dystrophin staining of tissue sections by immunofluorescence (Supp Fig. 1). Two individuals with in-frame mutations (Deletion E3–23 and Duplication E2–7) had symptom onset in by age 6 years, suggestive clinically of DMD rather than Becker Muscular Dystrophy, and both biopsies had detectable dystrophin by immunofluorescence, and the individuals were grouped with “Mild” severity based on their functional scores. Biopsies with > 2% in-frame *DMD* by RNAseq and with observed sarcolemmal staining of dystrophin by immunofluorescence were also categorized as DMD_DYS^lo^ and the remainder as DMD_DYS^−^. We find a strong enrichment of DMD_DYS^lo^ within the Mild group (*P* = 10^−5^), and they have lower plasma CK (*P* = 10^−5^) (Fig. 1f,g). All DMD biopsies had histologic appearance of dystrophinopathy, and all healthy biopsies had no history of muscle disease or recent injury and no histologic abnormalities. Mean age for healthy was 21.2 years, Mild 11.1 years, Typical 5.7 years, and Severe 5.2 years. Age of control biopsies was not a substantial influence on identified induced genes in DMD (Supp Fig. 2) ^19^.

In total, 134,943 nuclear transcriptomes were generated (22,492 from healthy, 37,480 from Mild DMD, 41,815 from Typical DMD, and 32,916 from Severe DMD, with an average of 4,217 nuclei analyzed per individual, and 873 genes per nucleus after removal of poor-quality data and doublets. Unbiased clustering of all nuclei identified 10 major cell classes in muscle tissue: myofibers, satellite cells, fibroblasts, myeloid cells, lymphoid cells, mast cells, adipocytes, smooth muscle cells, pericytes, and endothelial cells (Fig. 1a) with expected cell type-specific markers identifying each cluster ^3, 18^(Fig. 1b, and Supp Table 1). All major cell types were observed in each DMD group and the healthy group (Fig. 1c), but there were significant differences in muscle cellularity with decreased percentage of myofibers (*P* = 5×10^−4^) and pericytes (*P* = 5×10^−3^), and expanded populations of fibroblasts (*P* =2×10^−4^), satellite cells (*P* = 2×10^*−7*^), and myeloid cells (*P* =1×10^−5^) (Fig. 1d,e) in DMD relative to healthy. The Mild DMD group with detectable dystrophin partially returns towards healthy proportions of myofibers, fibroblasts, satellite cells, and myeloid cells (Fig. 1d,e, Supp Table 1). These data establish a correlation between disease severity and observed cellularity and gene set shifts.

### Dystrophic myogenic precursors accumulate in the satellite cell niche

Satellite cells (SCs) in DMD and healthy skeletal muscle clustered together by UMAP relative to other major cell populations (Fig. 1a) and were distinguished partially by expression of *PAX7, FGFR4,* and *CALCR* (Fig. 1b). Across all DMD samples, 8% of all muscle nuclei were SCs, representing a 2.9-fold expansion relative to healthy (Fig. 1e, *P*= 2×10^−7^). Although SCs of DMD and healthy clustered together relative to other muscle cells, re-clustering of SCs from DMD and healthy revealed substantial and consistent disease status differences (Fig. 2a-d). Healthy SC primarily tightly cluster as a single group (90% SC_1) and demonstrate significantly higher *CXCL14* expression relative to DMD SCs. Furthermore, healthy SCs (SC_1) have no evidence of cell cycling by gene expression, consistent with a fully quiescent state expected from uninjured healthy muscle^8 25^. By contrast, the SCs from DMD muscle separate into clusters SC_2A, SC_2B, and SC_2C, which maintain high expression of *PAX7*, *CALCR*, and *FGF4*, but do not overlap with the healthy SC_1 and are more diverse in their gene expression as shown by broader distribution in UMAP. Gene expression data suggest that few DMD SCs are quiescent based on an upregulation of *CCND2*, *CCND3*, and *SKP2*, consistent with exit from quiescence and proliferation (Supp Data Table 2). All three SC_2 subpopulations have higher *MET*, *MTOR* and *RPTOR* expression relative to healthy SC_1, consistent with SCs in DMD having exited from quiescence into at least G alert ^9,10^. Two less abundant populations within the SC cluster are labeled as SC_3 and SC_4, which downregulate *PAX7* and *MYF5* (Fig 2e), and likely reflect myogenic precursors that are transitioning toward myoblast differentiation. SC_3 upregulates *TOP2A* and *CCNB2* genes associated with increased proliferation, while SC_4 upregulates *MYOG* and *MYOD1,* and are likely differentiating toward myofibers. We perform pseudotime lineage tracing of DMD and healthy SC together to infer likely developmental trajectory, despite that the majority of healthy are SC_1 quiescent and the majority of DMD are SC_2 subpopulations. SC_1 could differentiate into a SC_2A like population which is then likely able to develop into SC_3, and then SC_4 creating a proposed healthy developmental trajectory (Fig. 2b), even though limited numbers of healthy are observed that are not in the main quiescent group. By contrast, lineage-based evidence suggests that the DMD-expanded populations SC_2B and SC_2C create an alternate trajectory from SC_2A and do not differentiate into SC_3 or SC_4. Given substantial expansion of immature myofibers in DMD, relative to healthy, there is evidence that some DMD SC can differentiate and contribute to immature myofibers (Fig. 2g-i). We propose that immature myofibers arise from DMD SC_2A, which are most abundant in Mild DMD, and the ratio of SC_2C to SC_2A increases with disease severity (Fig. 2c,d). We hypothesize that SC_2C represents ineffective myogenic precursors that cannot return to quiescence or properly commit to myogenic fate. SC_2C cells accumulate within the stem cell niche and may compete with other SCs, thereby disrupting regenerative capacity over time. SC_2B has a 70-fold induction of *FOS* (along with *FOSB, JUN, JUNB*, and *EGR1*) relative to SC_1 which may be due to signaling through several TNF receptor family members *TNFRSF21*, *TNFRSF1A*, *TNFRSF10B* or *TLR4* expressed in DMD SCs (Supplemental Table 2). Transient induction of *FOS* in response to muscle injury in healthy murine SCs ^26^ has been reported to drive satellite cells to expand into new muscle fibers through upregulation of *ART1* and other genes, though, our mouse *mdx*, human DMD and mouse/human healthy single nuclei data sets identified downstream targets including *ART1* that are only detected in myofibers lacking *PAX7* expression ^26^.

Gene set enrichment analysis of genes upregulated in each DMD SC population relative to healthy SC_1 identifies epithelial-mesenchymal transition (EMT) and myogenesis (Fig 2j and Supp Table 2), and SC_2B and SC_2C show significant enrichment in the term “TNF signaling through NFκB” (Fig. 2j). Both intrinsic satellite cell defects and extrinsic factors, including TNF signaling, may promote activation of cell cycle in DMD SCs and accelerate G1-S transition, leading to expansion of SCs while inhibiting differentiation/maturation ^11,12^. Further, *MET* upregulation in DMD SCs (3.2 fold) with engagement of SC-expressed *CXCR4* is predicted to inhibit differentiation and protect dystrophic SC from TNF-induced death, which we hypothesize contributes to accumulation of dysfunctional satellite cells in the dystrophic niche and limits the regenerative capacity of myofibers over time ^27^. These data support prior observations of an intrinsic stem cell defect in asymmetric division and show how an aberrant niche contributes to disruption of normal SC activation and differentiation ^8–10^ and provide an expression atlas of 9,684 DMD SCs for further analysis.

### Dystrophic myofiber gene expression reflects immune activation and myofiber death

Myofibers comprise the largest nuclei cluster (40–70% of all nuclei) and show high expression of myofiber genes such as *TTN* and *NEB* (Fig 1b). Since nuclei are purified “en masse” and computationally resorted based on gene expression, we cannot observe the full myofiber syncytium but rather organize the data into nuclei clusters. Type 1 and type 2 fiber clusters are distinguished by *MYH7* and *MYH1* expression, respectively, and immature fibers express *MYH3* and *MYH8* (Fig 2e) ^3, 18, 28^. An immature fiber type, expressing *MYH7*, and lncRNA *MEG3*, clusters separately from Type 1 immature DMD fibers and is termed here D_Type1_MEG3^hi^, is more abundant within Severe DMD (Fig 2g,h,i) and, thus, is correlated with disease severity. In Severe DMD there is a substantial increase of immature myofibers (17% of all myofiber nuclei, 17-fold higher than healthy muscle), and D_Type1_MEG3^hi^ (12% of myofibers and 296-fold increase). There is a relative loss of mature type 2 fibers relative to type 1 in Severe DMD, consistent with the specific vulnerability of type 2 fibers to death in DMD^3, 29^. Overall, Mild DMD muscle, which has some dystrophin expressed in myofibers, shows a trend toward normal (Fig. 2g,h,i).

Because of the expansion of immature and D_Type1_MEG3^hi^ fibers, and preferential loss of type 2 fibers (Fig. 2h,i) in DMD, prior bulk muscle gene expression studies could not distinguish differences in mature DMD myofibers from those due to the expansion of immature myofibers. To provide insight into how mature myofibers are affected in DMD, we restricted our comparison to type 1 or type 2 myofiber nuclei from Severe and Typical DMD relative to healthy. Type I and II myofibers were analyzed separately to generate a differential gene expression set for each type (Fig. 2i). We removed the immature and MEG3^hi^ (which appear to be a version of immature myofibers) from this analysis to avoid conflation with immature fibers. Gene expression differences of DMD versus healthy within mature myofibers types identified significant increases in 3,260 genes in type 2 and 1,864 in type 1 myofibers (Supp Table 2). Most genes upregulated in type 1 fibers are also increased in type 2 fibers (69.3% or 1,291 of 1,864 genes in type 1) indicating a similar transcriptional disease response in mature myofibers despite a greater loss of type 2 myofibers (Supp Table 2). Gene set enrichment analysis of upregulated genes highlights induction of innate and adaptive immune sensors, *TLR4*, TNFR family members and MHC and costimulatory genes (Fig. 2j), known to regulate inflammation and cell death through NFκB signaling, death domain signaling and activation of caspases and gasdermins ^30, 31, 32^. This is most apparent in DMD type 1 and MEG3^hi^ myofibers where: *TLR4* is upregulated 22 fold (P=10^−40^) and 24 fold (*P*=10^−114^), respectively; *TNFRSF10B*, 1.8 fold (*P*=10^−14^) and 2.7 (*P*=10^−62^) fold; *TNFRSF12A* 3.5 fold (*P*=10^−42^) and 2.5 (*P*=10^−51^) fold; and *TNFSFR21* 2.9 (*P*=.001) and 5.7 fold (*P*= 10^−25^). The TNFα receptor, *TNFRSF1A,* is constitutively expressed across healthy and DMD myofibers. Gene set enrichment set reveals significant enrichment terms including “TNF activation through NFκB”, “apoptosis’, “IL4 regulation of apoptosis”, “ITGB1 interactions”, “Epithelial Mesenchymal Transition (EMT)” and “myogenesis” (Fig. 2j and Supp Data Table 2). Of note TNFRSF1A, TNFRSF10B, and TNFSFR21 contain death domains that function in signaling cell death, while TNFSFR21 and TNFSFR12A do not, but all activate NFκB to some extent.

Analysis of the mature myofiber gene expression enabled an estimate of false discovery that may be driven by age, as our control cohort was young adults. To evaluate for this, we analyzed 288 strongly induced genes in DMD type 1 myofibers from published snRNAseq data of 7 DMD biopsies with a mean age of 3.5 years and 5 healthy males with a mean age of 8.33 years^19^ which are substantially younger than our control cohort and determined differential expression in the external dataset. We determined that only 5 of the 288 (1.7%) differentially expressed genes in our dataset were not observed in younger populations (Supp Fig. 2), suggesting that age is not a substantial confounder of our healthy versus DMD comparisons within myofibers. In the age ranges studied (3 to 22 years) there is not a substantial correlation of gene expression with age of identified differentially expressed genes by disease.

DMD upregulated genes show enrichment of pathways related to NFκB immune activation, inflammation, and/or apoptosis/pyroptosis (Fig. 2j and Extended Fig. 2a). Several genes are known to be upregulated by TLR4 agonist signaling and/or TNFRSFs including: MHC class I A, B, C and co-stimulators *CD80* (B7) and *CD276* (B7-H3) required for T cell stimulation and CTL/NK targeting; cytokines *IL15*, *IL32*, *TGFB1*/2/3 involved in driving fibrosis, and regulators of TNFR family and TLR4 programmed cell death and pyroptosis, *CYLD, RIPK2, NLRC4, NAIP, BIRC2, P2RY14, CASP2, CASP4, GSDMB, GSDME,* and lncRNA *MEG3 (CASP3* is constitutively expressed*)* ^33 34^ (Supp Table 2). Together, these data suggest that DMD myofibers upregulate immune triggers and are responding to TNFRSF family/TLR4 engagement to promote NFκB-driven inflammation and contribute to myofiber death. Upregulation of TLR4 protein expression on DMD myofibers ^34^ was confirmed by staining DMD and healthy frozen muscle sections from this same cohort (Fig. 2k,l). TLR4, TNFR family and GZMB mediated target cell killing, have been reported to induce activation of CASP3 and subsequent (or direct) cleavage of GSDME to GSDME-N activate fragment to effect apoptosis/pyroptosis (Fig. 2l). Additionally, Caspase-3 cleavage of β-spectrin is known to destroy the cortical cytoskeleton, further contributing to cell death ^35^. We find increased myofiber staining for both activated CASP3 and GSDME-N in DMD myofibers relative to healthy (Fig. 2l and Extended Fig. 2c,d). Of note, we find active CASP3 is localized to cytoplasmic and membrane staining patterns and associated with localized clearance of β-spectrin in affected myofibers^36^. These findings provide support for induction of myofiber programmed cell death in DMD, likely in response to TNFR family, TLR4 engagement, or GZMB mediated killing.

### Fibroblasts expand and diversify in DMD

Fibrosis is a major feature of DMD muscle histology. In muscle biopsies, fibroblasts/fibroadipogenic precursors (FAPs) expanded 2.3-fold in DMD muscle (Fig 1e, *P* = 10^−4^) and are distinguished from other DMD and healthy muscle cells by numerous fibroblast/fibroadipogenic markers (Supp Data Table 1) including *DCN*, *PDGFRA*, and *FAP* (Fig. 1b). We are unable to resolve fibroblasts or FAPs within this cluster and refer to this cluster as ‘fibroblasts’. Sub-clustering reveals ten related, but transcriptionally distinct, fibroblast subsets that are likely transdifferentiated within the dystrophic muscle, based on pseudotime lineage tracing (Fig. 3a-d). Known gene expression markers of FAPs, myofibroblasts and other fibroblast subpopulations and other genes that distinguish subpopulations are shown in Fig. 3d. FB_4 are not shifted in abundance in DMD and are most similar to adventitial pan-tissue fibroblasts and express *SEMA3C, DOCK4, CREB5* ^37^. FB_0, FB_1 FB_2, FB_3, and FB_6, are almost exclusively identified within dystrophic muscle (Fig. 3e), while FB_5 is the primary population within healthy muscle. Pseudotime lineage tracing suggests that FB_0, FB_1, FB_2, FB_3 and FB_6 are sequentially derived from FB_5 (Fig. 3b). DMD fibroblast populations were clustered by gene expression, and we calculated significant gene expression increases within each DMD specific population compared to healthy FB_5 (Supp Table 2). DMD fibroblast populations were significantly enriched for gene sets ‘epithelial to mesenchymal transition’ and ‘extracellular matrix reorganization’ indicative of tissue remodeling. FB_1 and FB_6 are most unique to DMD muscle, relative to the other fibroblast types. FB_1 expresses the highest levels of *ACTA1*/*ACTA2*, *TCF4, TCF7L2*, and *CTNNB1*, markers of “myofibroblasts”, as well as *IGF-1*, *TGFBs*, and several collagen-encoding genes (Fig. 3f) ^38, 39^ and was has gene set enrichment of terms of “epithelial-mesenchymal transition” (EMT) (*P*=10^−8^) and “extracellular matrix organization” (*P*=10^−8^) and “regulation of endothelial migration” (*P*=10^−4^). Leading models of DMD and normal muscle regeneration invoke a role for “myofibroblasts” in guiding myogenesis through the secretion of soluble factors which impact muscle regeneration and remodeling of the niche for repair and/or fibrosis ^25, 40^, and FB_1 is most similar to previously described myofibroblasts.

### FB_6 resembles THY-1^+^C3^+^ inflammatory tissue priming synovial fibroblasts in rheumatoid arthritis

FB_6 was most divergent from healthy FB_5 and was not observed in healthy muscle. Relative to other fibroblasts, FB_6, downregulated *CD34* expression but maintained *PDGFRA* expression and had significant gene set enrichments in inflammatory response (*P* =10^−10^), TNFα signaling via NFκB (*P*=5×10^−5^), IFNγ response (*P*= 4×10^−18^) and TGFβ signaling (*P*=10^−5^), complement. (*P*=0.047) and metabolic shift involving mTOR PI3kinase/AKT activation (*P* = 10^−4^), glycolysis (*P*=10^−4^), and negative regulation of programmed cell death (*P* =10^−3^), (Fig. 3f and Supp Table 2). In addition, FB_6-induced genes include 151 genes of an 891 gene expression signature that defines culture-induced “inflammatory tissue priming” fibroblasts which are also observed within inflamed synovium in rheumatoid arthritis (RA) (Fig. 3f, *P* = 2×10^−11^) ^41 42, 43^. Inflammatory tissue priming is a recently described mechanism of local innate immune memory, wherein repeated stimulation with TNFα or other proinflammatory stimuli leads to C3-dependent, metabolically driven, epigenetic reprogramming of tissue fibroblasts, rendering them hyper-responsive to restimulation with pro-inflammatory cytokines and resistant to cell death. This reprogramming is a potential mechanism that enables fibroblasts to persist in the niche and facilitates exaggerated inflammatory responses to subsequent stimuli ^44^. We propose that FB_6 is driving inflammation in DMD skeletal muscle and highlight a substantial enrichment of FB_6 overexpressed genes relative to other fibroblasts in dystrophic skeletal muscle (Fig 3 f,h,l,p). Within the FB_6-enriched inflammatory priming fibroblast gene set, we highlight genes within functional categories reflecting metabolic shift, TNFR family ligands with death inducing capacity, cytokine/chemokine signaling to other cells, multiple TLR4 agonists, and complement pathway effector protein gene expression (Fig. 3f).

To characterize FB_6 under dynamic conditions, we cultured primary patient-derived muscle fibroblasts from selected DMD muscles that had high proportions of FB_6 fibroblasts (by snRNAseq)(Fig. 3g). Fibroblasts were cultured with or without repeated TNFα stimulation. Gene expression response, measured by bulk transcriptomics, demonstrated TNFα-induced genes including C3 complement, CCL2 immune cell chemoattractant, tenascin C (*TNC*), and a significant enrichment of genes from the inflammatory tissue priming signature previously seen in RA (*P*=3×10^−4^)^44^ (Fig. 3g). Further, cultured DMD muscle-derived fibroblasts reflected the expression signature observed from snRNAseq data indicative of *in situ* human expression in DMD FB_6. Thus, cultured DMD fibroblasts are similar to FB_6 within DMD skeletal muscle (Fig. 3g) and can serve as a model to characterize requirements for inflammatory priming and effector functions. In keeping with the identified *in situ* FB_6 signature and synovial inflammatory fibroblasts, cultured DMD-muscle fibroblasts maintain responsiveness to TNFα, inducing genes within the Inflammatory Priming genes including cytokines/chemokines, TLR4 agonists, and complement effector proteins. Induced genes *CCL2*, *TNC*, *C3,* and other members of the Inflammatory Priming genes, are suppressed by co-treatment with an inhibitor of mTOR (rapamycin) or a bromodomain inhibitor (iBET151), reflecting requirements for metabolic reprogramming via mTOR and epigenetic remodeling in driving FB_6 maintenance and effector function (Fig. 3k,n,q).

### FB_6 expresses TNF family members predicted to trigger myofiber death and inflammation by binding TNFRs

FB_6 also expresses several TNF family cytokine genes including *TNFSF10*, *TNFSF12*, *TNSF14*, which are predicted to bind TNFR family members upregulated in DMD myofibers and other cell types. *TNFSF10* and *TNFSF14* are more abundant in FB_6 than healthy FB_5, and their receptors *TNFRSF10B* and *LTBR*, respectively, are upregulated on DMD myofibers. *TNFSF12* is expressed at low levels in all fibroblasts, but its receptor, *TNFRSF12A* is upregulated 5-fold in Severe DMD myofibers. *APP* is expressed across all fibroblasts, but its activating proteases *ADAM10* and *ADAM17*, are most upregulated in FB_6 and its TNFR binding partner, *TNFRSF21* is upregulated 8-fold in myofibers.

*TNFRSF10B* and *TNFRSF21* encode death domains involved in triggering cell death, TNFRSF21 also highly activates NFκB, whereas TNFRSF12A and LTβR lack death domains and primarily activate NF-κB, but can contribute to cell death in some settings. Consistent with some contribution of TNF receptor-mediated death driven by expression of ligands from FB_6, DMD myofibers upregulate apoptosis genes (Fig 2j) and activated CASP3 and GSDME-N protein (Fig 2l). We inferred cell-cell interactions of cytokines/chemokines induced in FB_6 with other cells in DMD muscle expressing receptors, which predicts significant interaction and signaling between FB_6 expressed *TNFSF10* and *TNFSF14,* and interacting receptors on myofibers and other cell types (Fig. 3o) ^45^. Of note, *TNFRSF1A* is constitutively expressed on healthy and DMD myofibers and encodes a death domain with high death and NFκB activating activity. Therefore, upregulation of TNFRFs and TLR4 on myofibers coupled with expansion of FB_6, which express ligands for these receptors in DMD, together with increased expression of activated death effectors in myofibers, predicts that FB_6 may function to directly kill myofibers, contributing to myofibers loss, in addition to orchestrating NFκB upregulation of pro-inflammatory effectors in myofibers.

### FB_6 expresses cytokines and chemokines predicted to influence recruitment and activation of multiple DMD intramuscular cell subsets.

Select chemokine/cytokine genes are induced in FB_6, including *CCL2*, a chemoattractant for recruitment of monocytes, macrophages, dendritic cells, and memory T cells to inflamed tissue, and is observed at higher levels in DMD plasma (Fig. 3m) and DMD muscle explant supernatants (not shown). Primary DMD fibroblasts stimulated with TNFα or IFNγ secrete CCL2 into culture supernatants and the combination is synergistic and can be suppressed with rapamycin, iBET151, and givinostat, an HDAC inhibitor that recently received FDA approval for DMD (Fig. 3k,n,q). These findings suggest some aspects of FB_6 response *in situ* may be driven by IFNγ, provide additional support for HDAC-mediated epigenetic reprogramming in establishing FB_6 functionality, and highlight a potential target of givinostat to modulate DMD disease severity

The set of genes encoding cytokines/chemokines produced in FB_6 versus healthy FB_5 in DMD were explored using literature and NicheNet to predict ligand-target links between interacting cells by analyzing upregulated cytokine/chemokine genes detected in FB_6 with gene expression of potential target cell interactions (Fig. 3o). These interactions suggest a central role for FB_6 within DMD muscle, predicting impact on many other intramuscular cell types through ligand-receptor interactions. CCL2 upregulation in FB_6 may drive recruitment of immune cells expressing the receptor CCR2 and CCL2 may interact with ACKR1 on endothelial cells to increase permeability and further invoke transcytosis^43^. HGF is highest and most induced in FB_6 and would be predicted to interact with MET on DMD SCs, suggesting a role for FB_6 in driving the pathogenic expansion of dystrophic satellite cells. FGF18 is upregulated in FB_6 and would be predicted to interact with FGFR4, which is specifically expressed on SC, and has been proposed to function in regulating satellite differentiation ^46^. We propose that the expansion of FB_6 in DMD muscle tissue contributes to cytokine and chemokine signals that impact the viability, expansion, development and effector activities of the multicellular muscle niche and is predicted to be a significant driver of muscle inflammation in DMD.

### FB_6 expresses and secretes TLR4 ligands.

We identified multiple TLR4 ligands (Fig. 3h) selectively upregulated in FB_6 *in situ*, including *TNC, THBS1, FN1, BGN,* and *HSPD1*^47, 48^, suggesting that FB_6 is a disease-specific source for TLR4 ligand engagement on other TLR4+ cells. Quantification of TLR4 ligands across all muscle resident cells reveals FB_6 as a primary source of: *TNC,* increased 73-fold *P*<10^−100^ in FB_6 relative to all other intramuscular cell types; *THBS1*, increased 22-fold *P*=10^−267^ (and 8.1-fold in DMD satellite cells *P*=10^−261^) ^49^; *FN1,* increased 2.3 fold *P*<10^−41^ and *BGN*, increased 42-fold *P*<10^−308^ in FB_6 and 13-fold *P*<10^−308^ in non-FB_6 fibroblasts versus non-fibroblast populations within dystrophic muscle. In comparison, *HSP90B1* is increased 2.1-fold in FB_6 (*P*=10^−20^), *HSPA4* is increased 1.3-fold in FB_6 (not significant), and *HSPD1* is increased 1.9-fold *(P*=10^−8^) in FB_6 compared to all other cell types, so are less specific to FB_6, rather upregulated in DMD muscle from multiple other cell types (Supp Table 2). Because of the observed induction of *TNC, FN1,* and *THBS1* expression in FB_6 in DMD muscle and their known role in myogenesis and fibrosis ^33, 37, 49^, we measured plasma levels of these proteins across our DMD cohort. Each is expressed at higher levels in DMD plasma (Fig. 3i,j), and we observe a correlation between *TNC* gene expression levels in DMD fibroblasts *in situ* with tenascin C protein levels in plasma (R = 0.334, *P* = 0.043). Thus, tenascin C in plasma is a biomarker of DMD and maps to FB_6 as the cell source. Cultured DMD fibroblasts constitutively secrete tenascin C (TNC), thrombospondin 1 (THBS1), and fibronectin (FN1) into the supernatant, and secretion is suppressed by co-treatment with rapamycin (Fig. 3k).

We next considered spatial colocalization of cell types and gene expression using published spatial transcriptomics data from *mdx* mice where^34^, unlike human dystrophic muscle, spatially discrete fibrotic lesions tend to develop to permit mapping of gene expression signatures to discrete pathologic locations within mdx muscle (Fig. 4). We identify inflammatory priming, apoptosis, TLR4 activation and FB_6 gene expression signatures within mdx muscle and selectively localized to fibrotic lesions and peri-lesion regions, while FB_1 expression signature is diffusely localized across dystrophic muscle, albeit at higher levels in dystrophic versus healthy muscle. Spatial colocalization of inflammatory fibroblast and expressed ligand gene expression highlights the likelihood of cell-cell interactions between FB_6 and other intramuscular cell types to control death signals, recruit immune cells, and remodel muscle at sites of most severe dystrophic pathology.

### FB_6 expresses genes encoding complement proteins in situ and secretes complement proteins into cultured FB_6 supernatants in response to TNFα/IFNγ

Comparison of genes upregulated in DMD FB_6 reveals multiple complement pathway genes, including *C3* (Fig. 3p). Both intracellular and secreted C3 have been implicated in metabolic shift involved in fibroblast inflammatory priming, and C4 secretion from cultured synovial fibroblasts is induced by IFNγ and suppressed by rapamycin ^44^. TNFα and IFNγ synergize to stimulate cultured DMD muscle fibroblast C3 secretion, while IFNγ induces C4 secretion, and both are substantially blocked by rapamycin (Fig. 3q). C3 secretion by FB_6 in DMD raises a potential mechanism, wherein C3 proteolysis to C3a and C3b contributes to increased inflammation, chemotaxis, and opsonization, and leads to the formation of membrane attack complexes with lysis of target cells ^50^.

### Endothelial cells show trends of reduced capillaries and expanded venous cells in DMD

Reclustering of snRNAseq data identified 4 classes of endothelial (*PTPRB*^+^) cells including lymphatic (*PROX1*^+^), venous (*ACKR1*^+^), capillary (*GPIHBP1*^+^), and arterial (*SEMA3G*^+^) endothelial cells (Fig. 5a-d). In DMD, there is a trend toward reduction of capillary endothelial cells (by 24%) and expansion of venous endothelial cells (by 37%), reflecting an alteration in vasculature of DMD skeletal muscle. Several gene set enrichment terms identified from differentially expressed genes between DMD and healthy endothelial cells, and DMD capillary endothelium(Fig. 5f). Gene set enrichment highlights extracellular matrix reorganization, angiogenesis, and cell adhesion. Indeed, VEGFA (upregulated in FB_6 and DMD myofibers) interaction with VEGFR2 (upregulated in DMD capillary endothelial cells) is predicted to mediate angiogenesis and endothelial cell permeability, survival and migration. Upregulated *MET* expression in venous and arterial endothelial cells could indicate responsiveness to HGF which is primarily expressed by FB_6 (Fig. 3o, Fig. 5f).

### DMD muscle-resident myeloid cells have signatures of trained immunity

Myeloid cells are known to be increased in DMD skeletal muscle, and our single nuclei data observed a 3-fold increase in DMD relative to healthy. To better describe DMD myeloid populations, we performed single-cell sequencing of DMD muscle immune infiltrates and joined them with myeloid cell signatures derived from snRNAseq data (Fig. 5d and Extended Fig. 5) We identified five populations of myeloid cells: a predominate mature macrophage population, termed MRC1^+^ three monocyte-like (mac/mono) populations, termed TAMo ^51^, PPARG^+^ and SPP1^+^, and dendritic cells (DC) (Fig. 5h-k). Calculated percentages show expansion of SPP1^+^ and PPARG^+^ mac/mono relative to MRC1^+^, DC and TAMo populations. Given the three-fold expansion of the myeloid population overall in DMD, all myeloid subsets increase in absolute number.

The MRC1^+^ subpopulation expresses *TNF, TGFB, TLR4,* and *CD86*, consistent with reports describing DMD muscle resident macrophage populations expressing mixed pro-inflammatory M1 and anti-inflammatory M2 markers associated with fibrosis, rather than repair ^1^. Of note, MRC1^+^ macrophages express complement receptors CR1 (c3b/c4b receptor) and C3AR1, whereas all mac/mono populations PPARG^+^, TAMo and SPP1^+^ express CR4 (ITGAX/ITGB2, iC3b receptor), establishing them as potential targets for C3 proteolytic effector fragments C3a and C3b (Fig. 5k and Supp Table 2)^52, 53^. While PPARG^+^ and TAMo mac/mono subsets express potent pro-inflammatory cytokines *IL1B* and *IL6*, *CD80* and *CD86* co-stimulator*s* and *C5AR1* complement receptors, only PPARG^+^ and SSP1^+^ express high levels of anti-inflammatory *IL10*. TAMo and PPARG^+^ express TLR4-regulated EGFR ligands, *AREG* and *EREG,* and transcription factors *CEPBP* and *FOSL2,* which drive their expression ^51, 54^. AREG and IL10 in combination promote muscle repair, perhaps implicating TAMo and/or PPARG^+^ in repair ^55^. *AREG* expression in mdx Treg has been implicated in promoting muscle regeneration and opposing fibrosis ^56, 57^ but has never been described before in mdx/DMD myeloid cells. AREG and EREG participate in both regenerative and fibrotic responses in other tissue settings ^58, 59^.

Trained immunity, a mechanism of innate immune memory relevant to myeloid cells, wherein TLR4-mediated epigenetic re-programming leads to hyper-responsiveness, has been identified in bone marrow-derived monocytes in mdx ^60, 61^. To explore the possibility that human DMD muscle resident myeloid populations are impacted by trained immunity, we compared induced gene expression signatures of myeloid cell populations in DMD muscle with published trained immunity expression signatures generated from human PBMC exposed to known trainers ^62^. We find enrichment of a common signature of trained immunity (innate training) in all three mac/mono populations (SPP1^+^ *P* =0.001, PPARG^+^ *P*= 0.02, TAMo *P*= 0.009, Fig. 5l). Each of these DMD populations have some specific enrichment of innate training signatures from known training stimuli, implying some degree of specialization (Fig. 5l).

Myeloid populations are well known to be stimulated, trained, and restimulated by TLR4 engagement, and FB_6 is a primary source of TLR4 ligands within DMD muscle. Thus, we sought to determine if myeloid populations *in situ* were TLR4-engaged by measuring the effect of TLR4 inhibition (with TAK-242) on gene expression within myeloid cells isolated from short term DMD muscle explants. We observe 252 genes significantly suppressed by TAK-242 in myeloid cells, that includes *IL6, IL1B, ADAM8* (Fig. 5m). This gene set shows enrichment of ‘TNFα signaling via NFκB’, ‘cellular response to LPS’, and ‘Toll-like receptor signaling pathway’ and others that indicate biologically relevant responses to TLR4 blockade in muscle explant myeloid cells (Fig. 5n). We infer that this set of genes is likely a TLR4 Activation gene expression signal in aggregate, and term this gene set ‘TLR4 Activation’ for simplicity to probe other cells for likelihood of TLR4 engagement *in situ* from snRNAseq data. Of note, the myeloid TLR4 Activation modules scores are highest in TAMo and PPARG^+^ monocytes which also have high immune training signature, likely reflecting evidence of immune training *in vivo*, but elevated in all myeloid subpopulations (Fig. 5o). Plotting the number of genes in the TLR4 Activation gene set suggests substantial TLR4 engagement also in type 1 myofibers, fibroblast subtypes, and endothelial cells (Fig. 5p) These findings support our model that TLR4 ligands engage TLR4^+^ myeloid cells *in situ* and support TLR4 signaling in TLR4+ non-myeloid cell subsets within DMD muscle. These data are consistent with ongoing TLR4 signaling to multiple muscle resident cells, likely by TLR4 ligands generated from FB_6. Further, identification of evidence of TLR4 Activation within FB_1, FB_2, FB_6 alongside FB_6 secretion of TLR4 agonist may reflect a feed forward loop which promotes and perpetuates development and inflammatory priming of FB_6. TLR4 agonist upregulation and FB_6 signature is observed in fibroblasts in an available smaller published dataset of 7 DMD and 4 healthy muscle biopsies and supports our findings (data not shown) ^19^. Leveraging published spatial transcriptomics and snRNAseq data from mdx mouse ^63^, we spatially localized gene expression signatures of FB_6, TLR4 Activation, immune training and myeloid populations to areas of mdx muscle that are fibrotic lesions (Fig. 6) indicating that primed FB_6, TLR4 ligands, TLR4 activation, and trained myeloid cells (Fig. 4 and 6) co-localize in mouse dystrophic muscle.

### DMD muscle tissue resident lymphoid cells, including CD8^+^ Tek, express granzyme K, TNFα, and IFNγ

To better characterize intramuscular T cells, we used cellular indexing of transcriptomes and epitopes (CITEseq) to simultaneously assess the expression of 33 T cell surface proteins with single-cell transcriptomics on CD45^+^ sorted muscle derived mononuclear cells. We identify the most abundant lymphoid cell populations as CD8^+^ central and effector memory cells (Tcm and Tem), followed by CD4^+^ and CD8^+^ memory precursors (CD8mp), CD4^+^ Tcm and Tem, γ/δ T cells, and NK cells (Fig. 7 a,b) and Supp Table 2). Comparison of lymphocyte populations identified by snRNAseq from DMD versus healthy muscle, Te, Tem and Tcm intramuscular T cells populations demonstrate 9.4-fold expansion relative to healthy (Fig. 7b). *ICOS* and *CD69* expression across multiple T cell subsets are markers of tissue residency, consistent with their roles in recruiting and retaining tissue-resident T cells ^64^. Smaller populations of Treg, MAITS, KIR2/3-DL1/2/3 T cells, and B cells are also present in the DMD muscle resident immune compartment (Fig. 7b) ^65^. *AREG* was not detected in Tregs, perhaps due to their limited abundance in our cohort. However, the relatively lower abundance of Tregs versus AREG^+^/EREG^+^ TAMo and PPARG^+^ populations leads us to infer that monocytes represent a primary source of AREG in DMD muscle.

Within the CD8subsets, *GZMB* (granzyme B), *GZMA* (granzyme A), *PRF1* (perforin) and *GNLY* (granulysin) are co-expressed in the CD8^+^ T effector (TEMRA) population, and a small percentage of Tcm and Tem, consistent with CTL killing activity. CTL, NK and γ/δ lymphocytes kill target cells through secretion of granzyme B and granzyme A, cleaving target cell gasdermins E and B, respectively, to effect pyroptosis ^30, 31^. Thus, we propose that upregulation of *GSDME* and *GSDMB* in DMD myofibers may prime them for killing by muscle lymphocytes (Fig. 9).

The bulk of the CD8^+^ Tcm and Tem are GZMK^Hi^GZMB^+^PRF1^Lo^GNLY^−^, resembling a newly described tissue-resident memory population, T effector K (Tek), expanded in inflammatory conditions, including aging, COVID and RA ^66, 67, 68, 69, 70, 71^. Tek are reported to have low cytotoxic activity and high cytokine production, which can be triggered by antigen dependent and independent stimuli, including IL15^64^.To characterize the tissue resident T cells, we compared snRNASeq data to a symphony reference dataset of synovial T cells obtained through Synapse (syn51281767) as part of the “Zhang Nature 2023 37938773” dataset under the Arthritis and Autoimmune and Related Diseases Knowledge (ARK) Portal which defined 24 tissue resident T cell populations by single cell gene expression, and all DMD T cell snRNASeq data were enumerated for abundance in DMD muscle using this naming scheme^59^. T-13 (CD8^+^GZMK/B^+^), T-14 (CD8^+^GZMK^+^ Memory), and T-15 (CD8^+^GZMB^+^ TEMRA) are identified in DMD with high confidence (Fig. 7c and Extended Fig. 7c) and are more abundant in DMD muscle. Additionally, the most induced population in DMD, T-13 (CD8+GZMK/B) and the T-14 (CD8+GZMK) had upregulation of RA_GZMK metacluster as defined by Jonsson et al from RA synovium^64^, (P=2.47×10^−4^) while T-15 (CD8+GZMB) had upregulation of the RA_GZMB metacluster, indicating a high degree of similarity of these key cells within DMD muscle and RA synovium. Within DMD muscle, there is an 8-fold expansion of CD8^+^GZMK/B^+^ T cells, a 2-fold expansion in CD8^+^GZMK^+^ memory and a 2.5-fold expansion in CD8^+^TEMRA (Fig. 7c). NK, γ/δ, and MAITs also express *GZMB*/*GZMK*, albeit at lower levels (Fig. 7 a,b), reflecting Tek similarity with innate-like T/NK/MAIT populations. While multiple T cells, and myeloid cells express *TNF*, and NK cells provide a potential source of IFNγ, CD8^+^Tek represent the primary and most expanded TNFα and IFNγ co-expressing cells, suggesting they may play an important role in perpetuating FB_6 priming and effector activity. (Supp Table 2).

Of note, most DMD muscle T cells, including CD8 T cells and NK cells express the receptor for IL15/IL15Rα, composed of IL2R common and IL2RB/IL2RG subunits. Further, *IL15* and *IL15RA* are upregulated ~2-fold when assessed across all cell types in DMD muscle. Thus, IL15/IL15α expressed on or released by dying myofibers, fibroblasts (highest on FB_6), endothelial, satellite cells and immune populations likely promote some T cell and NK expansion and/or activity. In keeping with this suggestion, IL15 is detected in supernatants of cultured muscle explants and in TNFα stimulated DMD fibroblasts (Fig. 7d).

IL15 is known to induce TCR independent CD8 memory bystander activity through upregulation of *KRLK1* (NKG2D) and *CXCR3*^66^. Both of these genes are induced across all DMD CD8 memory T cells in situ (Figure 7b,c and Supp Table 2), and the stress-induced ligand of NKG2D, *MICA*, is upregulated 3-fold in Severe DMD vs healthy across all muscle cells). Accordingly, diverse T cell/NK populations reflective of CD8 muscle T cells can be expanded with IL15 and/or IL18 in short term explant culture (14–21 days)(data not shown). Further, purified and expanded CD8+ T cells constitutively secrete TNFα, IFNγ, GZMK and GZMB into the supernatant (Fig. 7 e-h), in some instances more so in the presence of IL15, similar to RA synovial T cells showing similar capacities ^72, 73^. FACS analysis confirms that all CD8^+^ T cells express NKG2D, with per cell levels increasing after IL15 exposure (Fig 7). Taken together these data highlight the role of IL15 within DMD skeletal muscle driving pro-inflammatory signals creating a muscle resident T cell population less restricted to TCR engagement with a risk for a broader immune response within skeletal muscle.

In RA, granzyme K secreted by synovial Tek exacerbates inflammation through protease-activated receptor-1/F2R, upregulated in both RA synovial fibroblasts and DMD FB_6 ^66, 67, 74^. Indeed, GZMK activation of fibroblast surface PAR1/F2R has been reported to upregulate CCL2 and other proinflammatory genes in other inflammatory settings. Additionally, GZMK can also contribute locally to C3 cleavage and activation of downstream complement effector pathways through pathway in RA and inflammatory airway disease^70, 71^. By analogy, we propose that lymphocyte-secreted granzyme K may cooperate with FB_6 to promote the assembly of C3 convertase and cleavage of secreted C3, creating C3a and C3b effectors, capable of stimulating a wide range of functions including myogenesis, pyroptosis, chemotaxis, and phagocytosis through complement receptors upregulated on macrophage and mac/mono cells ^53^ and assembly of C5 convertase required for assembly of the membrane attack complex (MAC). MAC complexes (C5b-C9) have been observed in necrotic fibers in DMD and mdx muscle ^75, 76^. In support of these observations, spatial transcriptomics highlights colocalization of GZMK meta-cluster genes and complement family genes expressed by DMD fibroblasts (Fig. 8)

## Discussion

Here we provide a comprehensive description of intramuscular cell and gene expression profiles accompanying DMD muscle remodeling across a range of severity and dystrophin expression, creating a compendium of gene expression in DMD and healthy skeletal muscle at single cell/nucleus resolution. Simultaneous assessment of all intramuscular cell types allows quantification of cell dynamics and pathway analysis under circumstances of intact cell-cell communication, preserved in frozen muscle. We complemented these data with deeper assessment of purified muscle resident immune populations and follow-up experiments using frozen muscle, plasma, and cultured primary muscle fibroblasts and T cells from the same cohort. Dramatic tissue remodeling is captured with the replacement of myofibers and expansion of heterogeneous fibroblasts, dysfunctional and heterogeneous satellite cells, and myeloid cells contributing to a maladaptive response to muscle damage and relatively ineffective regenerative ability.

Muscles from individuals with a Mild DMD disease course were often expressing low levels of dystrophin and demonstrated gene and cell expression signatures intermediate between healthy and Severe DMD, enabling linkage of expression patterns with severity. These findings support growing evidence indicating that low levels of dystrophin expression are functionally significant ^3, 24^, the use of dystrophin as a surrogate biomarker for DMD accelerated approvals ^77^, and highlight the benefit of matched baseline dystrophin levels when using natural history comparator data sets as a surrogate for control cohorts ^23, 78^. Further, these data predict that similar analysis of DMD muscle from individuals treated with dystrophin replacement therapies will meaningfully reflect efficacy, and reveal mechanisms of action, as well as limitations. The scale of nuclei transcriptomic data makes it difficult to fully analyze all possible pathways alterations. Thus, we anticipate that the dataset, published in its entirety here, will serve as a valuable resource for both basic and drug discovery and integration with other datasets.

In several inflammatory conditions, C3^+^Thy1^+^ inflammatory tissue fibroblasts serve to provide local innate memory through inflammatory tissue priming, wherein repeated stimulation with proinflammatory stimuli, leads to fibroblast metabolic shift, epigenetic rewiring, and resistance to cell death, resulting in persistent localized inflammatory hyper-responsiveness to subsequent stimulation ^44 35, 79 80^. C3 secretion by synovial inflammatory tissue fibroblast has been linked to a novel pathway of complement activation ^66^. We describe an immune-responsive C3^+^Thy1^+^ fibroblast subpopulation (FB_6) unique to DMD, and not observed in healthy muscle, that expresses genes similar to a published inflammatory priming gene signature identified in synovial inflammatory tissue priming fibroblasts. In culture, these cells TNFα and IFNγ responsive, which is partially suppressed by inhibitors of metabolic and epigenetic shift (rapamycin, iBET151 or givinostat).

Analysis of potential DMD specific cell:cell interactions focused around FB_6 *in situ* and short term cultured DMD muscle fibroblasts in culture with FB_6 like characteristics, coupled with CITEseq analysis of purified muscle infiltrating cells, identified multiple potential immune sources of TNFα and IFNγ DMD, which are likely responsible for FB_6 metabolic shift, epigenetic rewiring and altered gene expression ^32, 81^. However, because the CD8+ Tek population is the most expanded, has the highest gene expression of *TNF* and *IFNG* and is co-localized with the FB_6 population in spatial data, we suggest it may play a key role in promoting fibroblast priming and effector activity. Further, we propose that the relatively select upregulation of TLR4 ligands/DAMPS and TNFSFs in FB_6 endows these cells with the ability to engage TLR4 and TNF family death receptors upregulated on DMD myofibers to promote death and inflammation, likely through apoptosis or pyroptosis, an inflammatory death pathway wherein processed gasdermins create pores of sufficient size for release of cytokines and induction of cell death ^31, 82^. DMD myofibers express high levels of *IL18*, *IL15*, *IL32,* and *HMGB1*, encoding pro-inflammatory cytokines that amplify inflammation and promote NK, γ/δ and CD8^+^ T cell development and activity. We identify three unique DMD intramuscular mac/mono populations with signatures reflective of TLR4-mediated trained immunity and a previously undescribed CD8 Tek GZMK^+^ memory T cell population associated with tissue inflammation ^57,66, 67, 68^.

Based on our findings, parallels with immune mechanisms operating in RA synovium ^41, 42^, recent identification of TLR4-mediated trained immunity in mdx, and published human training gene expression signatures ^60^, we propose a model wherein self-amplifying epigenetic mechanisms of innate memory operating in DMD FB_6 and mac/monos function to prime the niche for pathological immune processes which contribute to myofiber death, maladaptive muscle regeneration and fibrosis in DMD (Fig. 9).

The FDA has approved 8 disease-modifying therapeutics for DMD: targeting inflammation (deflazacort, vamorolone), broad epigenetic remodeling (givinostat), or rescuing/replacing dystrophin (four exon skipping antisense oligonucleotides and one AAV-mediated microdystrophin) ^83^. Corticosteroids suppress inflammation and slow progression, though they are far from curative. While dystrophin rescue/replacement therapies target the primary defect in DMD, their efficacy has been lower than expected. Additionally, rare adverse events in microdystrophin gene therapy have been associated with complement activation and expansion of dystrophin-reactive CTL ^83, 84, 85^. The potential for secretion of C3 by FB_6, coupled with the expansion of TeK, may predispose to complement-related adverse events, perhaps justifying intervention with C3, rather than C5 blockers, or directed suppression of FB_6 ^53, 86, 87^. These data are useful for re-interpretation of why many drugs have failed to demonstrate clinical efficacy in DMD. For instance, *CCN2,* upregulated on FB_6, encodes CTGF, which was proposed and tested as a therapeutic target in DMD through a specific anti-CTGF antibody, pamrevlumab (NCT02606136, NCT04371666, NCT04632940). Receptors, ITGA5 and ITGB2 and LRP are broadly expressed and upregulated in DMD muscle supporting a potential role of CTGF upregulation in DMD pathogenesis. However, the LELANTOS trial failed to detect clinical benefit, implying that a more complete understanding of drivers of fibrosis is essential for more effective anti-fibrotic therapies in DMD (NCT04632940). Similarly, blocking TNFα signaling, apoptosis/pyroptosis, or IL15/IL15a surface expression or secretion by DMD myofibers may prevent the expansion/activity of dystrophin or broadly reactive TEMR, Tek or NK cells which may exacerbate pathology. We propose that DMD fibroblast inflammatory priming and mac/mono trained immunity imprint the muscle niche; epigenetically instilling a memory to perpetuate the hyper-inflammatory DMD state and likely imposing a barrier to normal myogenesis and resolution of myofiber damage solely through dystrophin replacement. Significant research is now focused on identifying drugs to specifically block innate memory and adaptive immune effectors across multiple inflammatory conditions, and these may hold therapeutic promise for DMD ^41, 61^. The resource we have built in describing clinically relevant features of human DMD at single cell/nuclei resolution and mechanisms of myofiber death, inflammation, impaired myogenesis and fibrosis has enormous potential for identifying targetable pathogenic pathways, driving and validating drug discovery^88^.

## MATERIALS AND METHODS

### Human Subjects

27 Duchenne patients and 5 healthy volunteers were consented (UCLA IRB approvals 18–001366, 19–00090, 15–001919, 11–001087) for core needle muscle biopsy, peripheral blood draw and medical record review. All individuals had North Star Ambulatory Assessment (NSAA) close to the time of muscle biopsy or age at loss of ambulation or current age if over age 16 years old and ambulant. Some of the data were also obtained from medical record review.

### Muscle biopsies

All samples were obtained using a VACORA (Bard) vacuum-assisted core needle biopsy as previously described ^2, 47^. Each 125mg biopsy core was dissected into about 25mg pieces and processed for live cell culture as explained below or flash frozen in liquid nitrogen within tissue cassettes within 5 minutes of excision and stored in liquid nitrogen until RNA extraction or sectioning for histological examination.

### Nuclei extraction

About 3mg of flash frozen biopsy was prepared from 40 micron sections into 1.5ml RNAse free tube on dry ice, suspended in 0.5ml of an ice-cold solution of 0.2μm filtered 1% bovine serum albumin (BSA) in phosphate buffered saline (PBS) with 100U/ml of type IV collagenase (CAT: #07426, 100 mg from Stem Cell Technologies) and 0.5U/μl RNAse inhibitor (RNAse protector, Sigma Ref # 03335402001, Mannheim, Germany), and transferred to a glass dounce on ice. 7 strokes with A-gap dounce were followed by 7 strokes with B-gap, suspension was 70μm filtered and effluent centrifuged at 600g for 6 min at 4C. Nuclei pellets were resuspended in 0.5ml of 1% BSA in PBS with RNAse inhibitor (0.5U/μl) and DAPI (10μg/ml) and incubated at 4C for 1 hour. Stained nuclei were purified from debris by BD FACS ARIA II sorter using 70μm nozzle. Purified singlet nuclei were collected in 0.5ml 1%BSA in PBS with RNAse inhibitor (0.5U/μl), washed and filtered through 40um prior to library construction.

### Single nuclei library sequencing

10,000–20,000 nuclei were loaded from each sample onto 10x Chromium Single cell 3’ v3 for snRNASeq library construction and sequenced using Illumina Novaseq 6000 S2 2×50, following manufacturer guidelines (10x Genomics, Doc #CG000204) to target depth of 40,000 reads per nuclei library.

### Derivation of primary fibroblasts from muscle biopsies

20mg pieces of muscle were sterilely diced into 1–2mm pieces and digested with dispase (Worthington)(1.5u/ml) and collagenase (Worthington)(1000u/ml) at a 1:1 ratio at 37°C for 15 minutes. Cells were then passed through a 70μm mesh filter, centrifuged at 1200 rpm for 4 minutes, and resuspended in Nutrient Mixture F-10 HAM (Millipore Sigma), 20% FBS (Omega Scientific), and 1% penicillin/streptomycin. Following a 60 minutes incubation at 37°C, the cell suspension was transferred to another container to enrich for the myoblasts, while the attached cells were enriched for fibroblasts, and maintained in culture. A concentration of 5ng/ml of basic fibroblast growth factor (Peprotech) was introduced on day 3, with media replacement every 3 days.

### Histology/Immunofluorescence

Skeletal muscle tissue cross-sections were cut to 10 μM after equilibration at −22°C in a cryostat and then stored at −80°C until immunofluorescence or hematoxylin/eosin staining was performed. Slides were acclimated to room temperature and sections were circled using a hydrophobic barrier pen, fixed with 4% paraformaldehyde for 10 minutes, and permeabilized using 0.5% Triton-X in PBS for 10 minutes at room temperature. Sections were submerged in 3% BSA in PBS for 1 hour at room temperature and then incubated in primary antibody in blocking solution overnight at 4°C in a humidified chamber. Primary antibodies used were: CD284 (TLR4) Monoclonal Antibody (HTA125, eBioscience), Rabbit Polyclonal Caveolin-3 (ab2912, Abcam), Cleaved Gasdermin E (GSDME-N) (Asp270) (E8G4U) Rabbit Monoclonal Antibody (#55879,) Cell Signaling Technology Active Caspase‑3 Antibody (AF835, R&D systems), beta-spectrin (NCL-SPEC1, Leica). Sections were then incubated in secondary antibody Goat anti-rabbit IgG (H+L) Cross-Adsorbed Secondary Antibody, DyLight^™^ 550 (#SA5–10033, Thermo Scientific) and Goat anti-mouse IgG (H+L) Cross-Adsorbed Secondary Antibody, DyLight 488 (#35503, Thermo Scientific) in PBS for 2 hours at room temperature. Slides were mounted in Antifade Mounting Medium with DAPI (Vectashield, H-1200–10). Images were obtained using a fluorescent microscope and processed using Image J (release 1.53c). To quantify the signal of a given protein in the myofibers, cellpose was first used to identify each fibers, then a mask was created with ImageJ on the b-spectrin signal, followed by the measurement of the signal intensity of each protein of interest in each defined regions of interest ^89^.

### Isolating immune cell infiltrates

Immune cells were isolated from fresh muscle biopsy after mincing with scalpels and disaggregated against a 70μm mesh filter, incubated at 37°C, in a mix of 1:1 1000U/ml collagenase (Worthington) and 1.5U/ml dispase (Worthington) for 30 minutes to 2 hours depending on tissue quantity and degree of fibrosis, to create a homogeneous population of mononuclear cells, which were collected after passage through a 40 μm filter, centrifugation, and washing once with PBS. Cells were cryopreserved before analysis.

### RNA extraction

Adherent fibroblast cells were scrapped directly in Trizol (Thermo Fisher Scientific) and total RNA was isolated using the Purelink RNA mini-kit (Thermo Fisher Scientific). Frozen skeletal muscle (9 to 25mg) was homogenized on ice in 500μL of Trizol for RNA extraction using standard protocol^68^.

### Processing of blood and protein quantification of plasma by ELISA

Blood samples were collected in CPT tubes (362761, BD bioscience), and plasma was separated from cells. Plasma was aliquoted and stored at −80°C until analysis as batches. Plasma samples were thawed and diluted 1/100 in PBS to measure the levels of THBS1 (EHTHBS1, Life Technologies) and TNC (EH446RB, Life Technologies) or 1/100,000 to measure the levels of FN1 (NC2050589, Abcam) via ELISA kits.

### Assessment of TNC, THBS1, CCL2, C3, and FN1 secretion by cultured muscle fibroblasts

To assess for TNC, THBS1, CCL2, C3, C4 and FN1 on primary DMD fibroblasts (at p1–5), cells were cultured in triplicate and stimulated with or without TNFα (Millipore Sigma) at 10ng/ml plus or minus rapamycin (Selleckchem) at 40μM, IFNγ at 100ng/ml (peprotech), I-BET151 (MyBioSource) at 1uM, Givinostat at 100nM (Selleckchem), every other day for 3 days and stopped at day 4 when the cell culture supernatants were stored at −80°C until the subsequent assays were conducted. Cells were cultured in SMGM basal medium (Promocell c-23260) complemented with 15% FBS for the entirety of the assay. Cell culture supernatants were analyzed using C3 (ab108823, Abcam), TNC (EH446RB, Life Technologies), CCL2 (R&D system DY279), THBS1 (Thermofisher EHTHBS1), C4 (Abcam ab108825), IL15 (41702 pbl assay science) to measure their respective protein levels.

### Derivation of T cells from muscle chunks, in vitro stimulation assays and Analytical intracellular flow cytometry

T cells were expanded from an approximate 20mg piece of DMD muscle for 2 weeks with IL15 (100ng/mL) and/or IL18 (50ng/mL) in Nutrient Mixture F-10 HAM (Millipore Sigma), 20% FBS (Omega Scientific), and 1% penicillin/streptomycin. Lymphocyte-like cells expanded significantly during this expansion phase. For in vitro stimulation assay, expanded T cells were washed, replated and stimulated for 5 hours with no additional compound, Dynabeads^™^ Human T-Activator CD3/CD28 (Gibco) at a cell:bead ratio of 5:2, or Invitrogen^™^ eBioscience^™^ Cell Stimulation Cocktail (500X) or overnight with no additional compound, IL15, IL18 or both. GolgiPlug^™^ (BD Pharmingen) was used in some experiments. Before staining for intracellular flow cytometry, cells were first incubated with an anti-human FC for 15min at 4degrees (Fc Block, Unlabeled, Clone: 3070 (BD Pharmingen). Cells were then stained for membrane proteins: CD8 with Cd8b-PEcy7 (25–5273-42 life technologies and CD314 with CD314 (NKG2D) Monoclonal Antibody (1D11), PerCP-eFluor^™^ 710, eBioscience for 20 minutes at 4C. Data were acquired on a BD Fortessa analyzer using FACSDiva software. Compensation and analysis were performed using FlowJo 10.10.0.

### Assessment of GZMK, GZMB, TNFα and IFNγ on expended T cells

Supernatants of expanded T cells were used to measure the secretion of GZMK (Abcam, ab31436), GZMB (R&D system DY290605), TNFα (R&D system DTA00D), and IFNγ (Thermofisher ENEHIFNG5) by ELISA.

### Antibody labeling for feature barcode

Antibodies with unique barcodes were obtained from Biogen (Total seq C panel). Antibody titers were derived by estimation based on clone equivalences in terms of PE expression. Antibody clones with minimal (one log, eg TCR a/b) separation between isotype control and antibody staining received the lowest category of dilution, and clones with the highest separation (4 log, eg CD3) received the highest dilutions. Staining was performed as follows: 1) Each infiltrate sample was stained for a specific barcode to distinguish each human donor and simultaneously stained with an aliquot of our panel of antibodies tittered as described above. Prior to staining each cryovial was thawed and washed 1x with a DMEM media (20%FBS, 1X penicillin/streptomycin), resuspended in 1:1 human AB negative serum (MT35060CI, Corning) and left on ice for 30 minutes. Antibody was incubated on ice for 20 minutes, washed 4 times in individual microfuge tubes in 1 ml of PBS with 1% BSA, and resuspended and pooled into two tubes of 10 individual donors or less for FACS. CD45+ cells were purified using a FACS ARIA instrument with 70μm nozzle into 1ml of PBS plus 0.1% BSA with 0.5U/ml RNAse inhibitor per 1.5 ml collection tube.

### Statistics and reproducibility

Raw sequence data for all samples were processed using Cellranger 6.1.2 Mkfastq (formation of fastq files) and Count to generate gene expression matrices aligned to GRCh38–2020-A reference genome provided by 10x genomics, including intronic reads. Downstream processing was performed in R 4.2.2. Data was log-normalized and scaled, and predicted doublets were identified using DoubletFinder_V3 and removed from the dataset along with nuclei containing <200umi. Remaining nuclei were batch corrected using Harmony R package 0.1.1 ^69^ and UMAP clustered using variable genes identified in Seurat 4.3.0 R package and major celltypes were subsetted and reclustered to identify subpopulation ^70^. Subpopulations were identified using Seurat FindClusters program.

Differential gene expression analysis was performed using Seurat Findallmarkers (Wilcoxon Rank Sum test) to identify significant marker genes for each population as well as identify differentially expressed genes between DMD and healthy nuclei. Multiple sampling was corrected using Bonferroni correction.

Pseudotime lineage tracing was performed using Slingshot 2.6.0 on UMAP re-clusterings of individual major cell types to identify developmental relationships of related subpopulations. To determine the relatedness of highly similar cell types we used the Pseudotime analysis R package Slingshot 1.7.2 ^71^. The remaining clusters underwent UMAP clustering, (Seurat Run UMAP based on the Seurat FindVariableFeatures) for use in Slingshot analysis.

P-values for expansions and contractions of cell types between DMD and Healthy were calculated using a Mann-Whitney U test (non-parametric test for small samples sizes).

### Gene Set Enrichment Analysis (GSEA)

GSEA against publicly available databases were performed using Enrichr r package (version 3.2)^72^. Public databases include: GO_Biological_Process_2023, KEGG_2021_Human, MSigDB_Hallmark_2020, NCI-Nature_2016, BioPlanet_2019, and Reactome_2022. Custom Gene sets were generated from published data. “Inflammatory Tissue Priming” is the differentially expressed genes from comparison of MSUMSU vs MSU samples (GSE163749), performed using R package DESeq2 (version 1.42.1): “Inflammatory issue priming” signature was defined by genes upregulated in MSUMSU (adj. p < 0.05, log2(FC) > 5). “Immune training” gene set was derived from ^49^ and is listed in Supplemental tables 2c and 2e and are defined as all genes with adj. P < 0.05. TLR4

GSEA from custom gene sets was performed using fgsea package (version 1.28.0) in R, genes were preranked by log2(FC).

RNA extracted from muscle-derived fibroblasts was used for stranded poly-A pulldown library preparation and 50-bp paired-end sequencing at 40 million reads using the Illumina NovaSeq X Series sequencing platform (RNA-seq performed by UCLA TCBG). Reads were filtered and aligned using STAR 2.6.0c (ref_genome_hg19_gencodev19), and a gene-level read count matrix was generated using featureCounts. Differential gene expression analyses were then performed using the R package DESeq2 (version 1.40.2). Differentially expressed genes were selected using an absolute log2 fold change of ≥1 and an adjusted P value of ≤0.01.

Pathway analysis for Endothelial cells was performed with Metascape ^90^ with minimum overlap: 3, minimum enrichment: 1.5), using filtered DEG lists (log2fc>1) for all endothelial cells and vasculary subtypes (venous, capillary, arterial). Pathway analysis was not performed for the lymphatic subset as there were no upregulated DEGs. A curated list of pathways reaching significance (q-value < 0.05) is shown. Metascape was chosen over EnrichR for pathway analysis due to the relatively small number of enriched genes in the endothelial population

### Cell-Cell Communication

We applied the NicheNet model (1) to examine the intercellular communication in the previously normalized and annotated single-cell data. Ligands were identified as differentially expressed in Fibroblast 6 vs. Fibroblast 5 (log2FC >1, adjusted p-value < 0.05). Ligand activity was calculated with NicheNet v2 Area Under the Precision Recall curve (AUPR) to predict the activities of ligands in regulating differentially expressed genes (log2FC ≥ 1, p-value ≤ 0.1) in recipient. The gene set of interest consisted of genes that were both differentially expressed and present in NicheNet v2 ligand–target matrix. The background gene set included all other genes expressed in the receiver cell type that were present in the ligand–target matrix. The ligand-receptor interactions were then manually filtered to only include the most biologically relevant pairs such that each cytokine/chemokine ligand connects is paired with one receptor. Furthermore, NicheNet uses existing knowledge of signaling networks to predict the effects of each ligand-receptor binding event on the downstream gene expression of a set of target genes that are differentially expressed (log2FC ≥ 1, adjusted p-value ≤ 0.05). The regulatory potential scores (weights) quantify the strength of evidence for each ligand–target relationship based on the integrated signaling and regulatory network strength. All analyses were performed using NicheNet (v2.0) with pre-built human prior knowledge networks. Visualization was performed using the circlize R package, with ribbon opacity scaled by ligand activity (AUPR) and radial bar height scaled by summed regulatory weight.

## Supplementary Material

Supplementary Files

This is a list of supplementary files associated with this preprint. Click to download.

- ExtendedFigure24.06.262.19pdf
- ExtendedFigure3.Fibrolasts3.20.26.pdf
- ExtendedFigure5.Myeloid3.20.26.pdf
- ExtendedFigure7.Lymphoidcella3.20.26.pdf
- SupplementalFigure14.06.26.pdf
- SupplementalFigure23.20.26.pdf
- Supptable1Natlmm12.12.25.xlsx
- Supptable2Natlmm4.06.26.xlsx

List of Supplementary Materials:

Supplemental Table 1: Cell type Identity and Proportions

Supplemental Table 2: Differentially expressed genes and gene set enrichment analyses

Extended Figure 2

Extended Figure 3

Extended Figure 5

Extended Figure 7

Supplemental Figure 1

Supplemental Figure 2

## Figures and Tables

**Figure:**
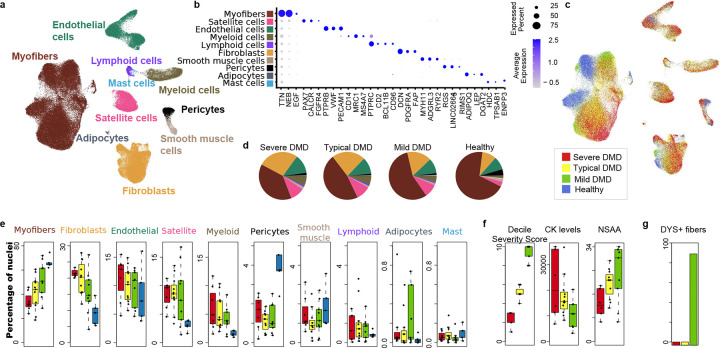


**Figure:**
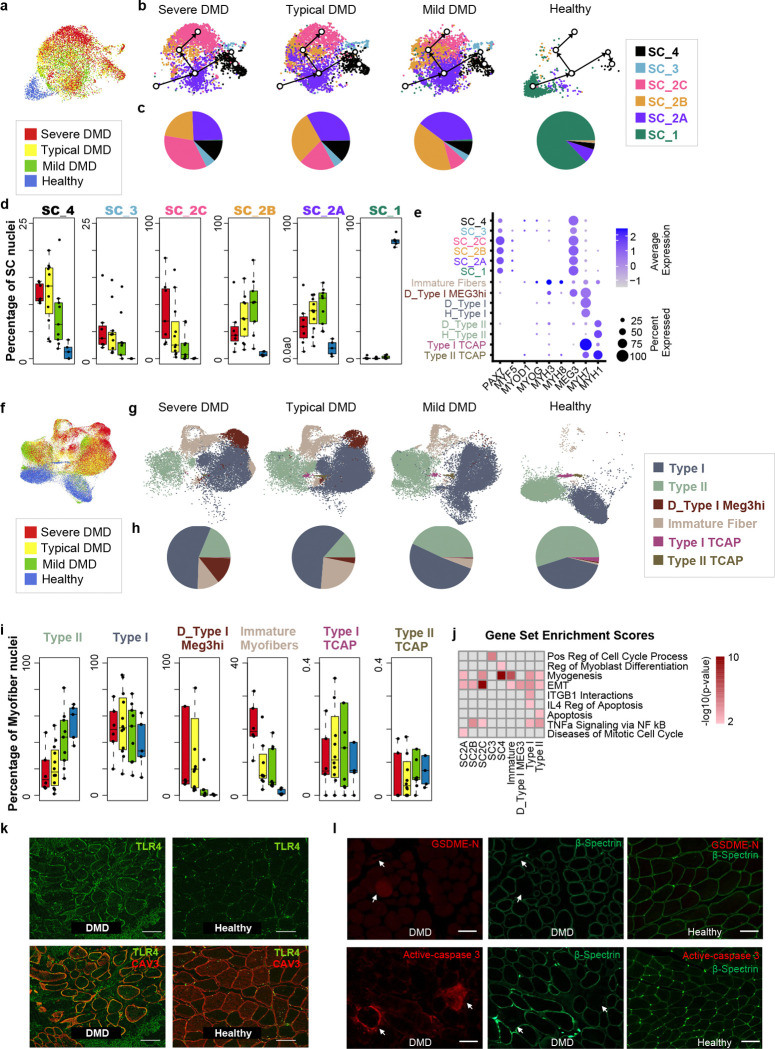


**Figure:**
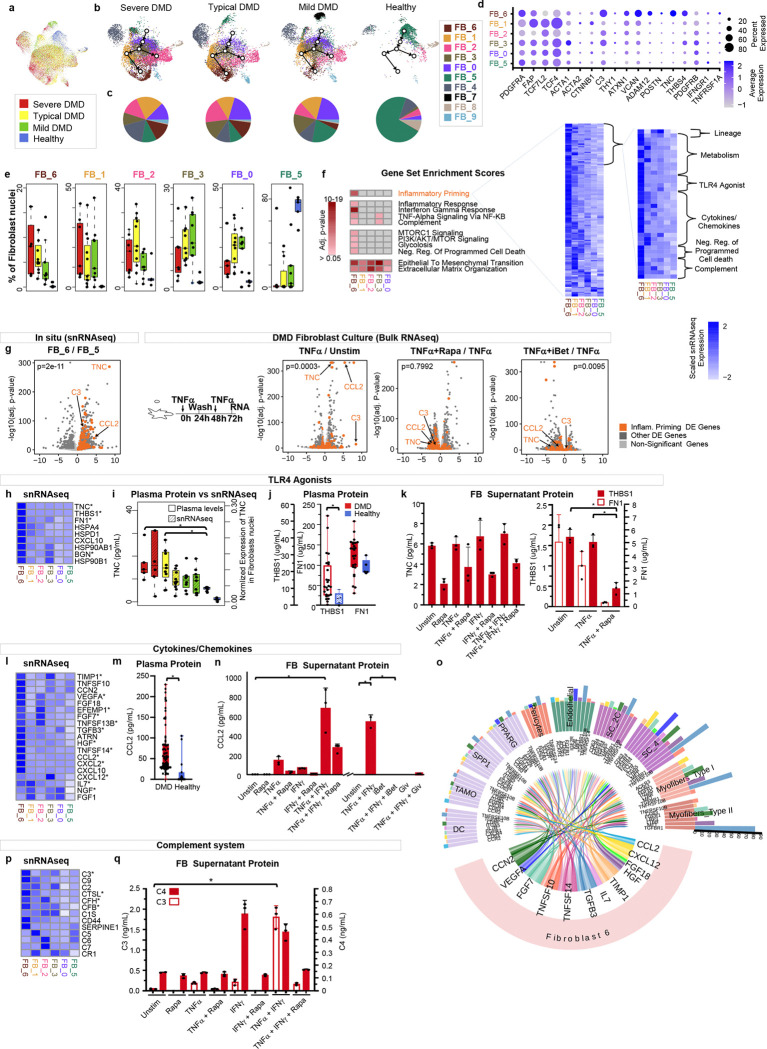


**Figure:**
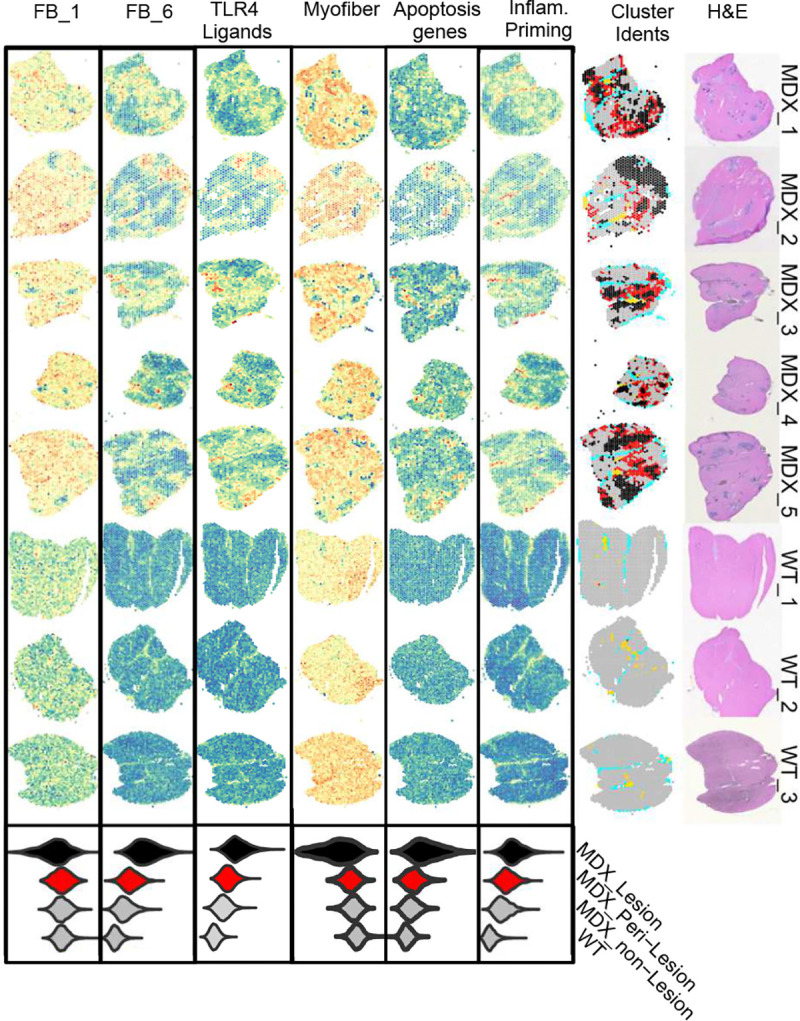


**Figure:**
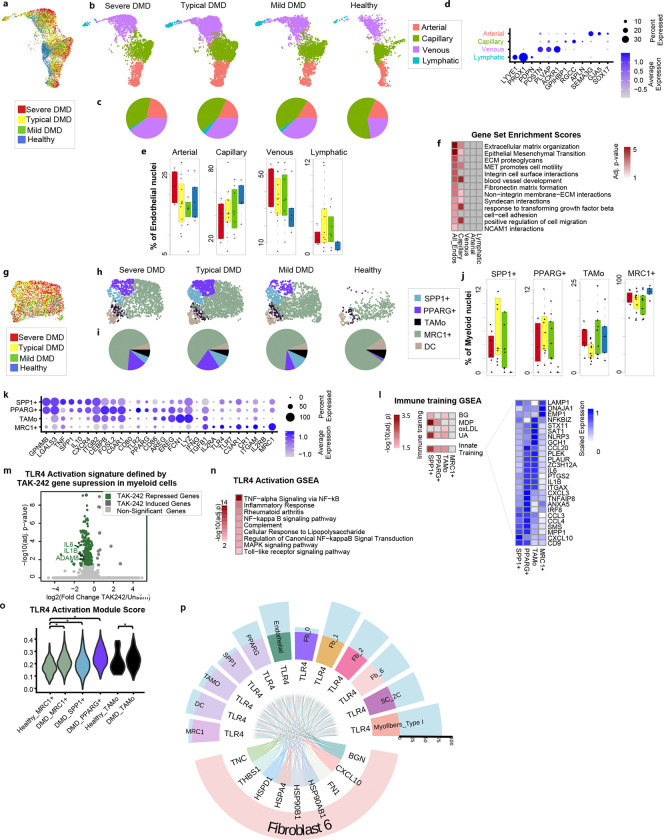


**Figure:**
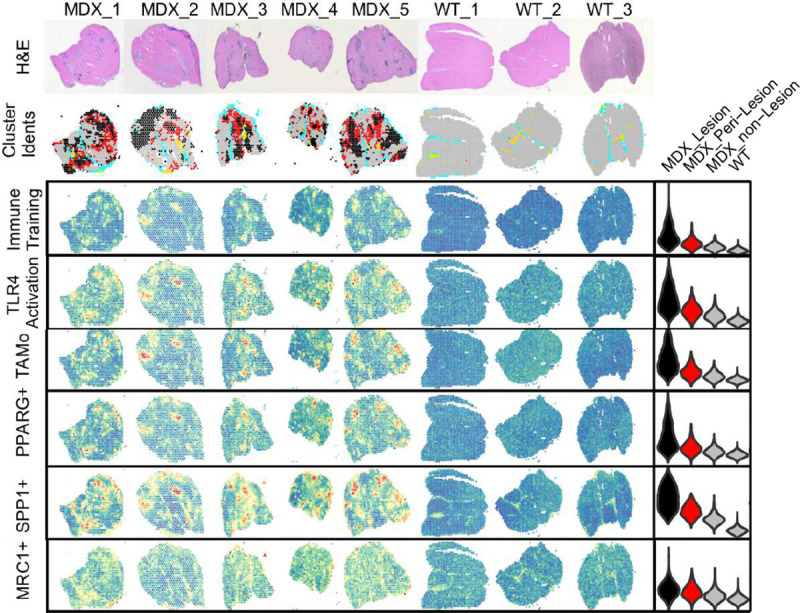


**Figure:**
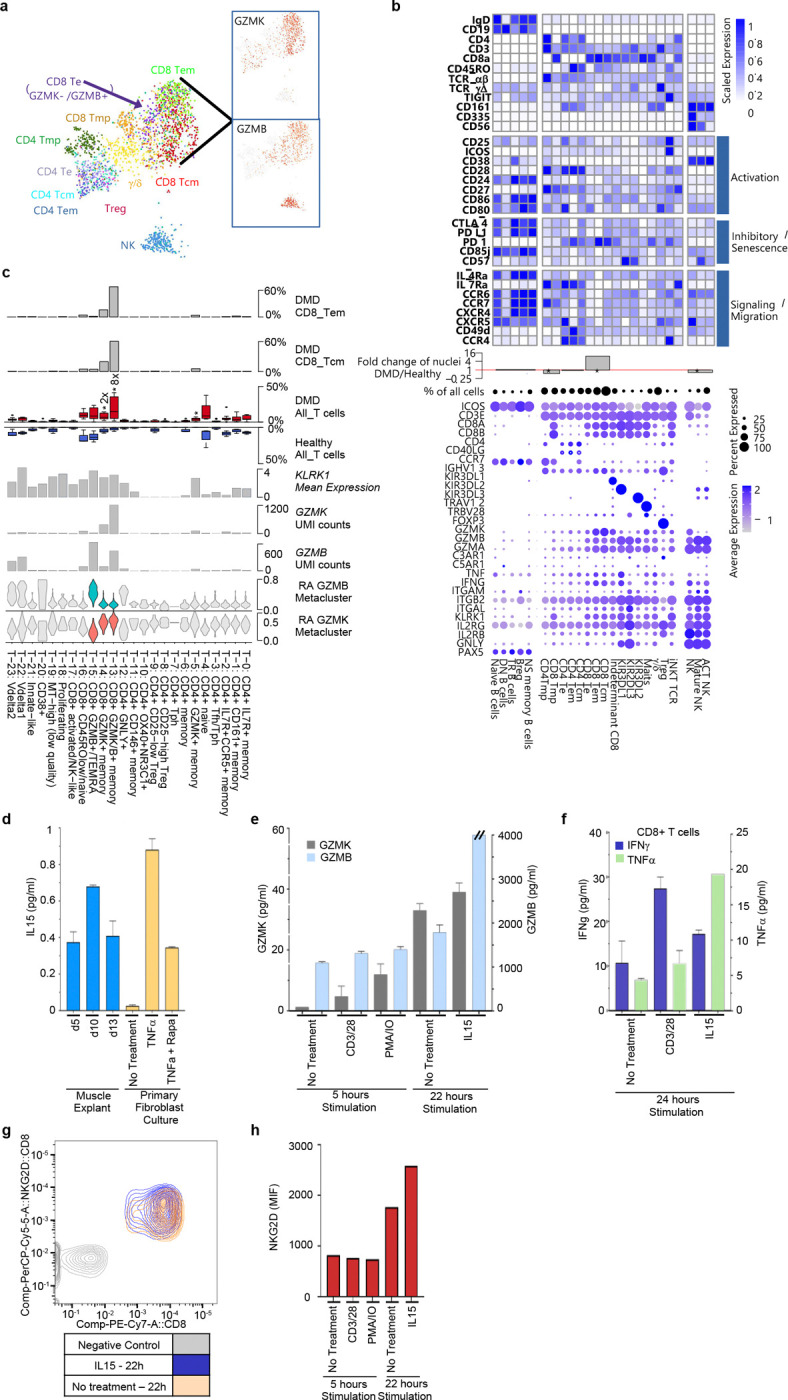


**Figure:**
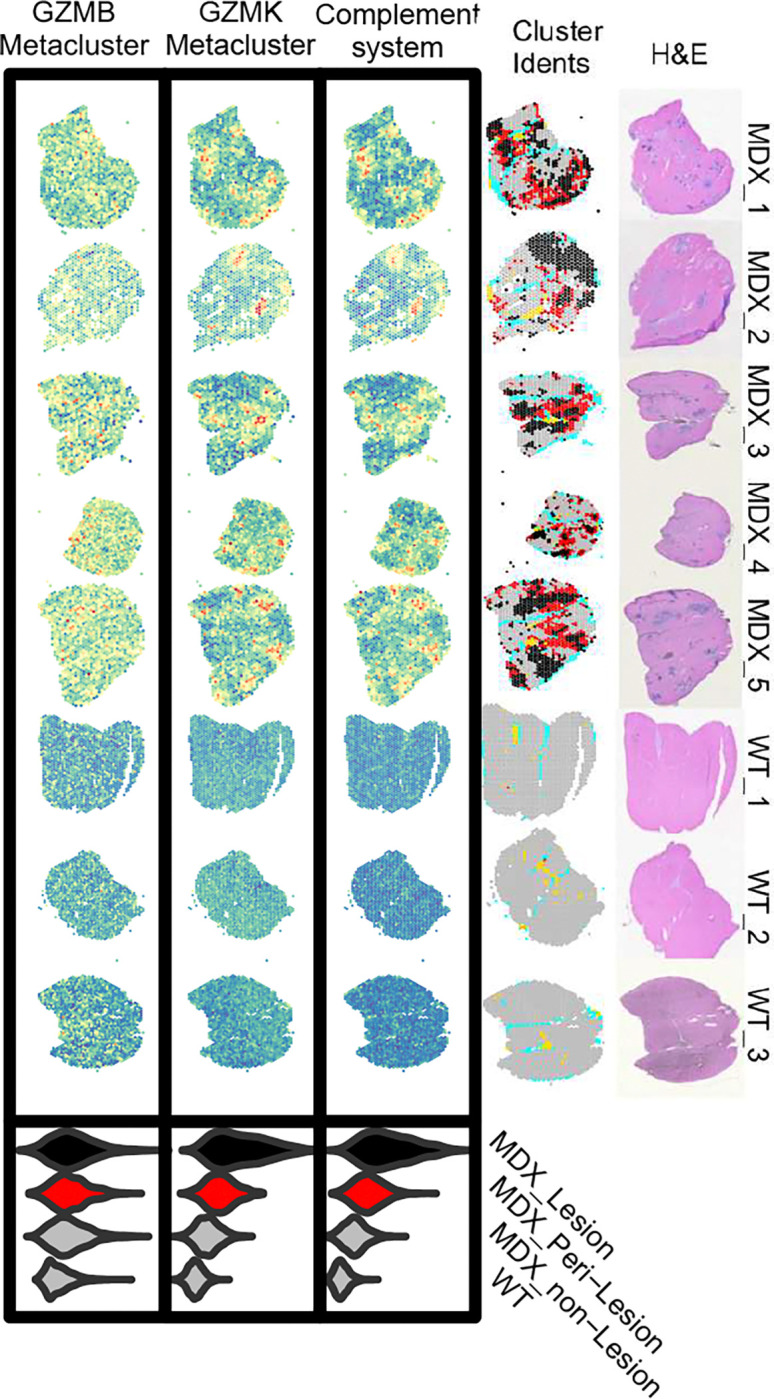


**Figure:**
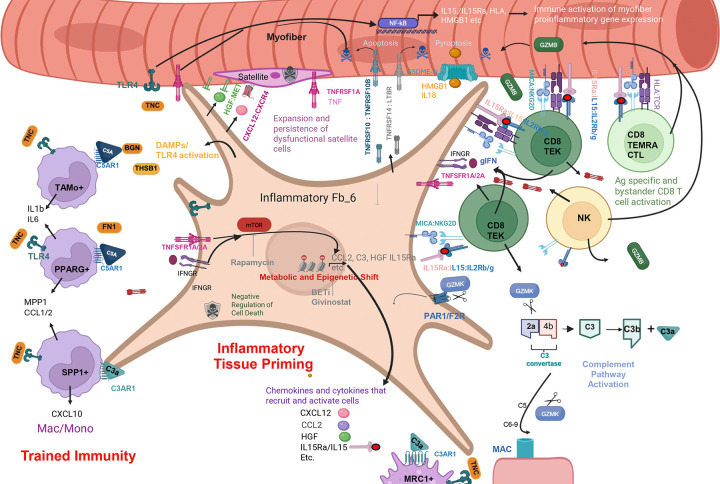


## Data Availability

All short read data is available in NCBI SRA under accession number PRJNA1151723. (Reviewer link: https://dataview.ncbi.nlm.nih.gov/object/PRJNA1151723?reviewer=ebgbscigpumnkmiloskn854dt6) Data derived from public databases: Immune training DOI: 10.1172/JCI147719, Inflammatory Priming DOI: 10.1016/j.immuni.2021.03.003, MDX spatial transcriptomics DOI: 10.1073/pnas.2221249120
